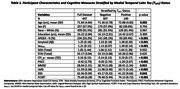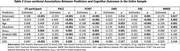# Exploring the Cross‐sectional Association of Mood Symptoms with Subjective and Objective Cognition in the A4 study

**DOI:** 10.1002/alz70857_104379

**Published:** 2025-12-25

**Authors:** Idris Demirsoy, Kellen K. Petersen, Bhargav Teja Nallapu, Richard B. Lipton, Ali Ezzati

**Affiliations:** ^1^ Usak University, Usak, Turkey; ^2^ Department of Neurology, Washington University School of Medicine, St. Louis, MO, USA; ^3^ Technical University of Delft, Delft, Zuid‐Holland, Netherlands; ^4^ Albert Einstein College of Medicine, Bronx, NY, USA; ^5^ University of California, Irvine, Irvine, CA, USA

## Abstract

**Background:**

Subjective cognitive impairment (SCI), a potential precursor to measurable cognitive decline, is a known predictor of Alzheimer's disease (AD). Mood symptoms, including depressive and anxiety symptoms, negatively impact both subjective and objective cognitive measures, even when accounting for tau and amyloid pathology. This highlights a critical yet underexplored area in understanding how mood symptoms independently influence cognitive outcomes beyond established AD biomarkers. This study examines the cross‐sectional relationship between mood symptoms and subjective (Cognitive Function Instrument, CFI) and objective (Preclinical Alzheimer Cognitive Composite, PACC) cognitive measures, while accounting for tau and amyloid burden.

**Method:**

We analyzed 447 participants from the A4 study, all of whom underwent tau PET and amyloid PET. Depressive symptoms were assessed using the Geriatric Depression Scale (GDS) and anxiety symptoms were assessed using the State‐Trait Anxiety Inventory (STAI), respectively. Multiple linear regression models were used to examine the cross‐sectional associations between mood symptoms and cognitive outcomes, adjusting for age, education, amyloid and tau burden (as measured in the medial temporal lobe; tau_MTL_) at baseline.

**Result:**

Higher levels of depressive symptoms were significantly associated with poorer subjective cognition (CFI = 0.047, *p* <0.001) and objective cognition (PACC = ‐0.099, *p* = 0.025), even after accounting for tau and amyloid pathology. Anxiety symptoms showed no significant associations with either CFI or PACC scores. Tau burden (tau_MTL_) was independently associated with lower PACC scores (=‐0.145, *p* = 0.001), while amyloid burden did not show significant associations with either cognitive measure.

**Conclusion:**

Depressive symptoms are strongly associated with both subjective and objective cognitive measures, independent of tau and amyloid pathology, highlighting the importance of addressing mood symptoms in individuals at risk for AD. Anxiety symptoms, in contrast, appear to have minimal influence on cognition in this cohort. These findings underscore the value of incorporating assessments of mood symptoms into AD research to refine early detection strategies and intervention approaches.